# Mikulicz's Operation

**Published:** 1890-03-15

**Authors:** 


### MIKULICZ'S OPERATION.

In Frieburg University Museum there is an excellent specimen of this operation, not generally known in England. It consists in removal of the distal portions of the tibia and fibula, together with the astragalus and os calcis. Union then subsequently taking place, the patient is left with a very serviceable foot, being able to walk on tip-toe.

